# Identification of *Aspergillus* at section and species levels by artificial intelligence-based microscopic morphology image recognition

**DOI:** 10.1128/jcm.00012-26

**Published:** 2026-02-27

**Authors:** Meng Tan, Zhe Guo, Yanyi Wang, Xinyi Xu, Wei Cao, Zhaoyang Liu, Chuanhao Jiang

**Affiliations:** 1 Department of Laboratory Medicine, The Second Xiangya Hospital, Central South University 620422, Changsha, Hunan, China; 2 Department of Laboratory Medicine, Hunan Provincial Rehabilitation Hospital, Changsha, China; 3 Clinical Molecular Diagnostic Center of Hunan Province, Changsha, China; University of Utah, Salt Lake City, Utah, USA

**Keywords:** convolutional neural network, deep learning, *Aspergillus*, morphology, image identification

## Abstract

Rapid and accurate identification of *Aspergillus* species in clinical microbiology laboratories is crucial for aspergillosis diagnosis and antifungal therapy. However, traditional methods still face challenges in distinguishing phylogenetically related species due to their morphological similarities. This study presents FungalNet, a deep learning model integrating ResNet-50 architecture with Focal Loss algorithm, specifically designed to enhance feature extraction for *Aspergillus* identification. A total of 12,000 high-resolution images were obtained from lactophenol cotton blue-stained slide preparations under a 100× oil immersion objective, among which 311 images were excluded through a novel quality control approach combining fivefold cross-validation and expert manual review. The performance of four deep learning models (FungalNet and three established models) for identifying *Aspergillus* species and sections was evaluated using the remaining 11,689 qualified images. FungalNet demonstrated superior classification performance, achieving overall accuracies of 98.45% and 97.85% at the section and species levels, respectively. These results indicate that FungalNet shows significant promise for rapid and accurate identification of *Aspergillus* species. With further optimization and multicenter validation, this tool could potentially be integrated into routine diagnostic workflows to enhance the efficiency and reliability of fungal identification in clinical settings.

**IMPORTANCE:**

This study integrates microscopic morphology identification with deep learning to address the challenge of accurate *Aspergillus* species identification. Twelve clinically isolated *Aspergillus* species belonging to eight different sections were included. From touch-tape slide preparations with lactophenol cotton blue staining under a 100× oil immersion objective, 11,689 qualified images were collected and analyzed using FungalNet (our proposed model) along with three established models (GoogLeNet, ResNet-50, and Xception). The results showed that FungalNet demonstrated superior performance in *Aspergillus* identification, achieving the highest classification accuracy at both section (98.45%) and species (97.85%) levels. Given its rapid turnaround time and cost-effectiveness, this AI-based image analysis approach shows promising potential for the rapid and accurate identification of *Aspergillus* species in clinical microbiology laboratories.

## INTRODUCTION

*Aspergillus* species are commonly found in the environment but can also be important opportunistic pathogens. Recent studies have demonstrated a progressive increase in invasive aspergillosis incidence (1). These infections predominantly pose a significant threat to immunocompromised populations, including patients with hematologic malignancies, acquired immunodeficiency syndrome, recipients of bone marrow and solid-organ transplants, and individuals undergoing immunosuppressive therapies (2, 3). It is noteworthy that such infections have also been observed in patients without traditional immunocompromising conditions, including those with severe influenza, novel coronavirus (SARS-CoV-2) infection, and chronic obstructive pulmonary disease (4–6). Given the high risk of morbidity and mortality associated with these groups, the rapid and accurate identification of *Aspergillus* species is crucial for the timely diagnosis and effective antifungal therapy.

Traditional morphological identification remains indispensable in clinical microbiology laboratories. The identification of *Aspergillus* species typically requires comprehensive examination of fungal colonies through both macroscopic and microscopic analyses, with particular attention to their distinctive morphological characteristics (7). However, morphological identification is a time-consuming process that relies heavily on the expertise of microbiologists. It is typically identified only at the genus or complex level owing to the morphological similarities among various *Aspergillus* species. In addition, molecular methods and matrix-assisted laser desorption/ionization-time-of-flight mass spectrometry (MALDI-TOF MS) have been increasingly employed in hospitals with greater financial resources for the identification of fungi (8, 9). While these two emerging methods have been shown to improve species-level accuracy, they are still subject to several practical limitations. Molecular methods need specialized expertise, labor-intensive procedures, and high operational costs, while MALDI-TOF MS is greatly limited by the complicated extraction procedures, as well as the inadequate spectra database (10, 11). These limitations highlight the urgent need to develop rapid, accurate, and cost-effective alternatives for *Aspergillus* species identification.

In recent years, artificial intelligence (AI) has significantly reduced reliance on manual intervention, contributing to the advancement of laboratory medicine (12). As a specialized branch of AI, deep learning has demonstrated considerable potential for clinical microbiology applications (13). This technology uses multi-layered neural networks for automated feature extraction and has been successfully applied to the automated interpretation of Gram-stained blood cultures (14), the detection of intestinal protozoa in trichrome-stained fecal specimens (15), and the morphological classification of bacterial vaginosis (16).

Convolutional neural networks (CNNs) are a leading tool in deep learning, drawing inspiration from the biological neural networks of the animal visual cortex (17). CNN has been widely applied in various computer vision tasks such as image classification, facial recognition, and object detection (18, 19). In some preliminary reports, CNN models have also been successfully applied to the morphological identification of *Aspergillus s*pecies. For example, Huang et al. developed five deep learning models to classify six *Aspergillus* species using a data set comprising 4,108 microscopic images captured from lactophenol cotton blue-stained slide preparations under 40× magnification (20). Among these models, Xception achieved the highest overall accuracy of 84.5%. Tsang et al. captured a total of 6,867 images of four common *Aspergillus* species grown on agar plates using digital cameras or smartphones (7). Three CNN models were subsequently trained and tested on these images. The results demonstrated that ResNet-18 achieved the highest performance, with an overall accuracy of 99.35%. Additionally, Ma et al. developed an automated system based on deep learning to differentiate seven *Aspergillus* species with a data set consisting of 8,995 macroscopic images (21). These images showing conidiophore and colony morphology were acquired using a dissecting microscope/stereomicroscope platform. Ultimately, the system achieved an overall accuracy of 98.2%. Despite the high overall classification accuracy demonstrated by these studies, their clinical applicability remains constrained due to the inability to incorporate phylogenetically related *Aspergillus* species, which exhibit highly similar morphological characteristics.

Moreover, Jing et al. collected 398 strains covering 39 mold taxa and developed the XMVision Fungal AI system by using 101,596 high-quality images, ultimately achieving an overall accuracy of 93.00% (22). Although the study incorporated a number of phylogenetically related *Aspergillus* species, the identification was limited to the complex level, with difficulty distinguishing similar species like *A. versicolor* and *A. sydowii*. This limitation is clinically significant because accurate species-level identification is crucial for effective patient management (23). Cryptic species within the same complex share high morphological similarity with common taxa but often exhibit distinct antifungal susceptibility patterns. For example, species within the *A. fumigatus* complex, such as *A. lentulus*, *A. udagawae*, *A. thermomutatus*, *A. felis*, and *A. fischeri*, are less susceptible to antifungal drugs than *A. fumigatus* (24–26). Similarly, within the *A. niger* complex, *A. tubingensis* typically exhibits greater azole resistance than *A. niger* (27). These discrepancies highlight the risks of misidentification, which can result in inappropriate therapy and poor clinical outcomes.

## MATERIALS AND METHODS

### *Aspergillus* species and strains

In this study, a total of 64 *Aspergillus* strains, as an initial *Aspergillus* resource, were consecutively identified by the microbiology laboratory of the Second Xiangya Hospital of Central South University between November 2020 and April 2024. These 64 *Aspergillus* strains included 12 species belonging to 8 sections. All strains were subcultured on potato dextrose agar plates and incubated at 28°C for 3 to 5 days prior to identification. Genomic DNA was extracted from pure cultures, and three loci—the internal transcribed spacer (ITS), β-tubulin (BenA), and calmodulin (CaM)—were amplified and sequenced using Sanger sequencing. The obtained sequences were compared with reference sequences in the NCBI database using BLAST and subsequently uploaded to GenBank. Species identification was based on a sequence identity of ≥98% with >95% query coverage. The corresponding GenBank submission number and detailed molecular identification results, including all identification discrepancies observed across the three loci, are summarized in Table S3.

### Slides preparation and image acquisition

**Fig 1:**
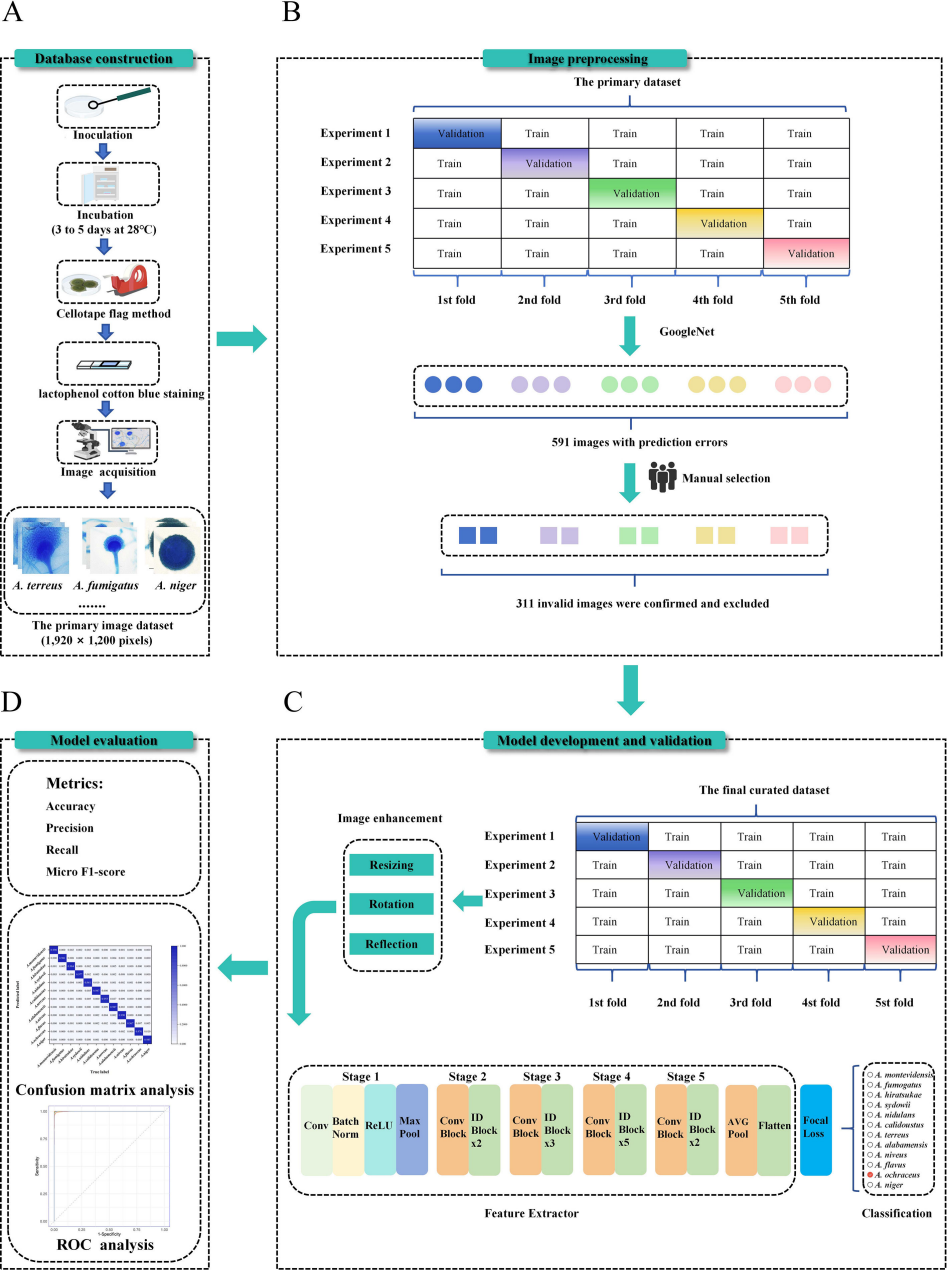
The workflow of the FungalNet model and the overall study design. (**A**) Image database construction of 12 *Aspergillus* species in the Second Xiangya Hospital of Central South University. (**B**) Image preprocession that integrates fivefold cross-validation and manual selection by microbial experts. (**C**) Development and validation of the FungalNet model. (**D**) Analysis methods and evaluation metrics for four models.

Specimen slides were prepared by lactophenol cotton blue staining and the cellotape flag method. These slides were initially inspected under a microscope to guarantee the quality and typical aspect of each slide. Additionally, the image data set was manually acquired using an Olympus BX53 microscope (Japan) equipped with a camera featuring a 1/1.8-inch sensor under 100× oil immersion objective. This study acquired a total of 12,000 microscopic images, with 1,000 images captured for each species. For each slide, approximately 30–40 images were captured, with an individual pixel size of 0.15 mm × 0.15 mm and an image resolution of 1,920 × 1,200 pixels. For different *Aspergillus* species, varying numbers of slides were prepared. For instance, for species such as *A. fumigatus, A. flavus, A. niger*, and *A. terreus* with sufficient strains, approximately 3–4 slides were prepared for each strain. In contrast, for some species like *A. alabamensis, A. hiratsukae, A. calidoustus*, and *A. niveus,* which include only a single strain, 30–40 slides were prepared. Ultimately, a total of 1,000 images were generated for each *Aspergillus* species to ensure adequate images for training and validation. All images were saved as TIFF format directly from the imaging device. The detailed workflow is presented in Fig. 1A.

### Image preprocessing

In this study, a fivefold cross-validation method was employed for image preprocessing by a CNN model called GoogleNet. Firstly, the image data set is split into five equal folds randomly, with each fold holding approximately the same number of images. Subsequently, four folds were used for training, while the remaining fold was used for validation on GoogleNet. This process was repeated five times, with each fold serving as the validation data set once, and the average performance of the training and validation data sets was calculated for the GoogleNet. Finally, all misclassified images were collected and reviewed by three professional clinical microbiologists. If at least two experts agreed that the misclassification was attributable to poor image quality, the corresponding images were excluded from the data set. The remaining images were then used for training and validation. The complete workflow is depicted in Fig. 1B.

### Image enhancement

A set of preprocessing techniques was employed for image augmentation, which involves applying random image transformations to generate additional training samples. These techniques help prevent overfitting and enhance the overall performance of the algorithm. The selected augmentation methods included resizing, rotation, and reflection. Moreover, all training images were uniformly resized to 418 × 418 pixels to achieve an optimal balance between computational efficiency and preservation of image detail.

### Development of the FungalNet model

In this study, we proposed FungalNet, a fungal image classification model based on ResNet-50 and the Focal Loss algorithm to handle severe class imbalance (28–31). We used Focal Loss to improve the multi-class classification performance on underrepresented categories. Focal Loss is an enhancement of categorical cross-entropy that dynamically scales the loss of well-classified examples to focus more on difficult, misclassified samples. The categorical cross-entropy loss is given by


$$H(p,\;q)=-\sum_{\text{i}\text{=1}}^{\text{n}}p_{i}logq_{i}.$$

Where *n* is the number of classes, $p_{i}$ is the true probability distribution (typically one-hot encoded), and $q_{i}$ is the predicted probability from the Softmax output. Since only one $p_{i}$ is non-zero and log⁡(0) is undefined, the Softmax ensures that all $q_{i}>0$. It should also be noted that cross-entropy is not symmetric, H(p, q)≠H(q, p). The Focal Loss for a single sample is defined as


$$L=-\alpha_{t}(1-p_{t}{)}^{\gamma }\;\text{log}(p_{t}),$$

where γ is a prefixed positive scale value and


$$p_{t}=\left\{ \begin{array}{l} P\;\;\;\;\;\;\;\;\;\;\;if\;y=1\\ 1-P\;\;\;otherwise \end{array}\right.,$$

$$\alpha_{t}=\left\{ \begin{array}{l} \alpha \;\;\;\;\;\;\;\;\;\;\;if\;y=1\\ 1-\alpha \;\;\;otherwise \end{array}\right..$$

This introduces two hyperparameters:

- α: a weighting factor to balance positive and negative classes. In our experiment, α = 0.25 yielded the best results.
- γ: a focusing parameter that reduces the loss contribution from easy examples. A higher γ value increases focus on hard samples. We found that γ = 2 works best in this study.

### Model training for *Aspergillus* identification

FungalNet, a proposed model, as well as three other established models (GoogLeNet, ResNet-50, Xception), were used as our CNN models. The development process involves several steps. To begin with, an input image was fed into the model, which passed through several convolution and pooling layers. Subsequently, each successive layer takes the output of the preceding layer as its input and is designed to extract increasingly complex distinguishing features from the images. At the end, the final obtained features are activated by a Focal Loss function to obtain the final network prediction result, which is represented as a probability vector spanning 12 classes. The predicted class corresponds to the class with the highest probability value. The entire process is illustrated in Fig. 1C.

### Model evaluation

In this study, the model’s performance was evaluated using a fivefold cross-validation approach, with the average score of the five folds reported as the final performance metric. Four evaluation metrics (accuracy, precision, recall, and micro F1-score) were used to assess the performance of four deep learning algorithms at both the section and species levels. Class-specific calculations were conducted for multiclass image classification using a one-versus-all approach. Furthermore, to prevent potentially misleading results, we adopted the micro F1-score instead of the macro F1-score for these multiclass tasks, given that an approximately balanced data set was collected across all *Aspergillus* species. The definitions and corresponding calculation formulas of the four metrics are presented as follows:

1. Accuracy: the proportion of correctly classified images out of the total number of images.
2. Precision: the proportion of true positive predictions among all positive predictions for a specific class.
3. Recall (sensitivity): the proportion of true positive predictions among all actual positives for a specific class.
4. Micro-F1 score: a performance metric used in multi-class classification tasks that calculates the F1-score by aggregating contributions from all classes (i.e., summing up true positives, false positives, and false negatives across all classes) and then computing a global F1-score.
5. Specificity: the proportion of true negative predictions among all actual negatives for a specific class, measuring a model’s ability to correctly exclude non-target species.

$$\text{Accuracy = }\frac{\text{TP}}{\text{(TP+FP+TN+FN)}},$$

$$\text{Precision = }\frac{\text{TP}}{\text{(TP+FP)}},$$

$$\text{Sensitivity = }\frac{\text{TP}}{\text{(TP+FN)}},$$

$$\text{Specificity = }\frac{\text{TN}}{\text{(TN+FP)}},$$

$$Micro-F1=\frac{2\times Precision\times Sensitivity}{(Precision+Sensitivity)}.$$

In addition to quantitative evaluation metrics, we also employed receiver operating characteristic (ROC) curves to visualize model performance. Furthermore, confusion matrices were generated for the classification of 12 *Aspergillus* species and eight sections, allowing a detailed assessment of prediction accuracy across the four CNN models. This approach is considered to be a convenient method for model optimization.

## RESULTS

### Database construction

A total of 12,000 high-resolution images were collected from 64 *Aspergillus* strains of 12 species across eight sections, with approximately 1,000 images acquired for each species. The distribution of these 64 strains is summarized in Table S3. The top four *Aspergillus* strains were *A. fumigatus* (15, 23.44%), *A. flavus* (13, 20.31%), *A. terreus* (12, 18.75%), and *A. niger* (10, 15.63%). In addition to these clinically common *Aspergillus* species, several less-encountered species like *A. montevidensis, A. alabamensis, A. sydowii, A. calidoustus, A. niveus, and A. hiratsukae* were also included (10, 15.63%).

### Image preprocession

To further enhance the quality of the entire image data set, this study adopted a CNN model (GoogleNet) with a fivefold cross-validation approach to analyze the primary image data set, achieving an average training accuracy of 95.04% and 591 wrongly predicted images. Three senior clinical microbiologists then manually reviewed these incorrectly predicted images, and 311 low-quality images of them were confirmed and excluded from the data set. The reasons for deleting these 311 images are summarized in Table 1. Of those images, 44.05% were eliminated due to the lack of typical *Aspergillus* characteristics, especially the absence of typical conidial heads. About 31.51% of the images exhibited unclear *Aspergillus* structures, because they were either out of focus or over-stained. Another 14.79% of the images were discarded due to improper brightness (overexposure or darkness), while the remaining 9.65% of the images contained obvious bubbles. After this quality control step, a total of 11,689 images remained, constituting the final curated image data set for further analysis.

**TABLE 1:** Detailed information of the 311 images that were excluded

*Aspergillus* species	Unclear structure^*a*^	Atypical morphology^*b*^	Conspicuous bubbles	Improper brightness	Total
*A. flavus*	23	22	1	7	53
*A. fumigatus*	6	11	3	7	27
*A. nidulans*	12	22	3	6	43
*A. sydowii*	8	10	0	2	20
*A. terreus*	15	20	3	9	47
*A. niger*	3	19	1	2	25
*A. ochraceus*	10	17	0	5	32
*A. montevidensis*	1	3	1	0	5
*A. niveus*	2	4	0	3	9
*A. alabamensis*	6	11	1	0	18
*A. hiratsukae*	1	4	3	2	10
*A. calidoustus*	11	8	0	3	22
Total	98	151	16	46	311

^*a*^ Images were out of focus or over-stained.

^*b*^ Images only displayed detached hyphae, spores, or images showed distorted *Aspergillus* vesicles.

### Comparison before and after image preprocessing

After image preprocessing, GoogleNet exhibited a slight performance improvement on the final curated image data set, with the model’s overall accuracy and precision increased by 0.35% and 0.32%, respectively (Table 2).

**TABLE 2:** Performance comparison of the GoogleNet model in *Aspergillus* species classification before and after data preprocessing

Model	Before data preprocessing				After data preprocessing			
	Accuracy (%)	Precision (%)	Recall (%)	Micro F1-score (%)	Accuracy (%)	Precision (%)	Recall (%)	Micro F1-score (%)
GoogleNet	95.01	95.07	94.93	95.00	95.36	95.39	95.25	95.32

### Comparative analysis of model performance

#### Classification of *Aspergillus* at the section level

**Fig 2:**
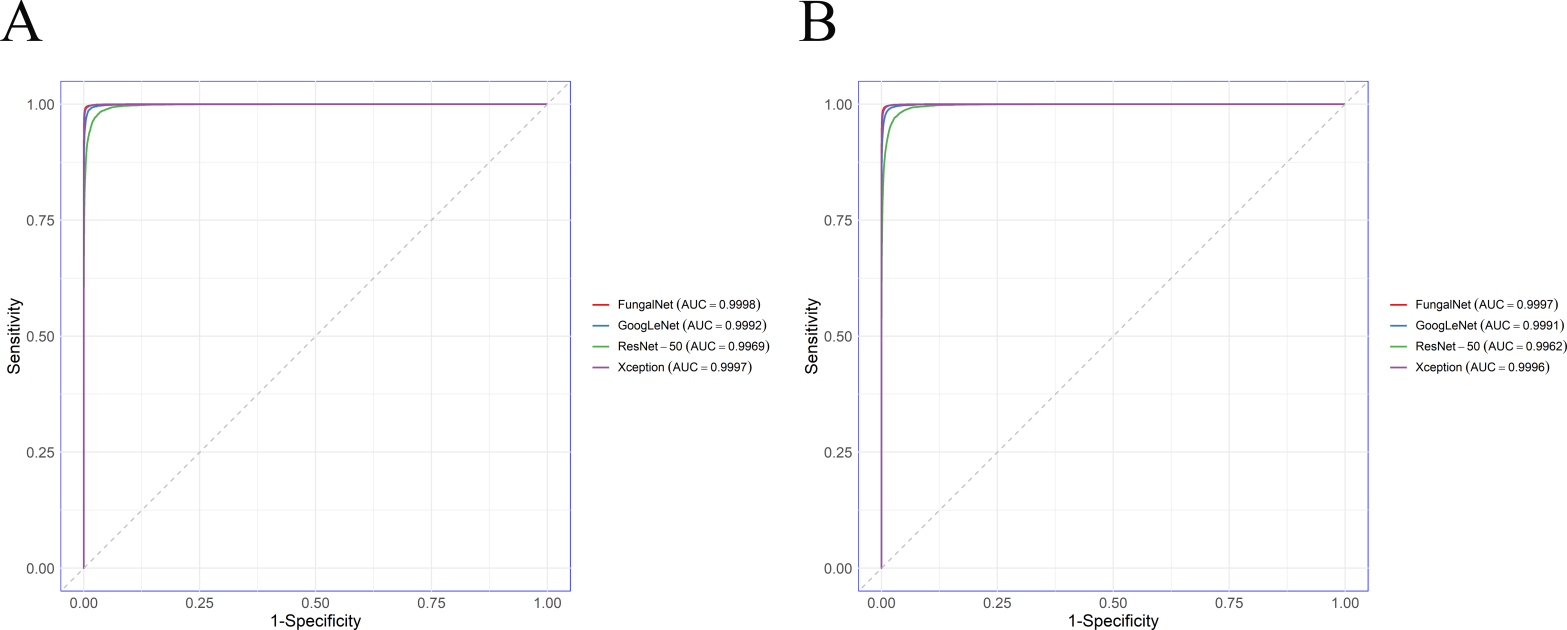
Comparison of ROC curves via the AUC values for four CNN models at the (**A**) section level and (**B**) species level.

**Fig 3:**
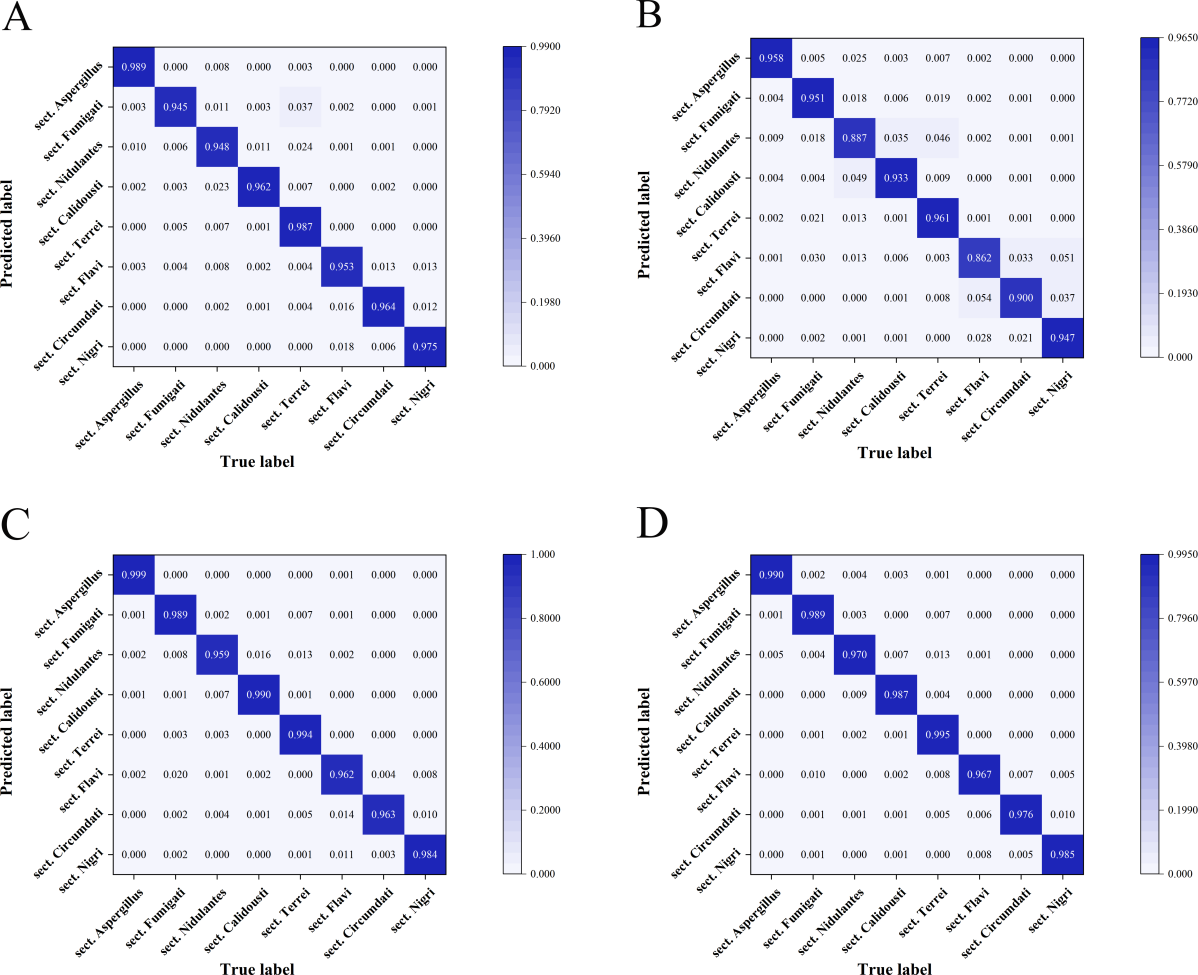
Confusion matrices of four CNN models for *Aspergillus* at the section level (eight sections). Each row represents an instance in the true class, and each column represents an instance in the predicted class. The diagonal line shows the prediction accuracy of the CNN model for different *Aspergillus* sections. (**A**) GoogleNet, (**B**) ResNet50, (**C**) Xception, (**D**) FungalNet.

**Fig 4:**
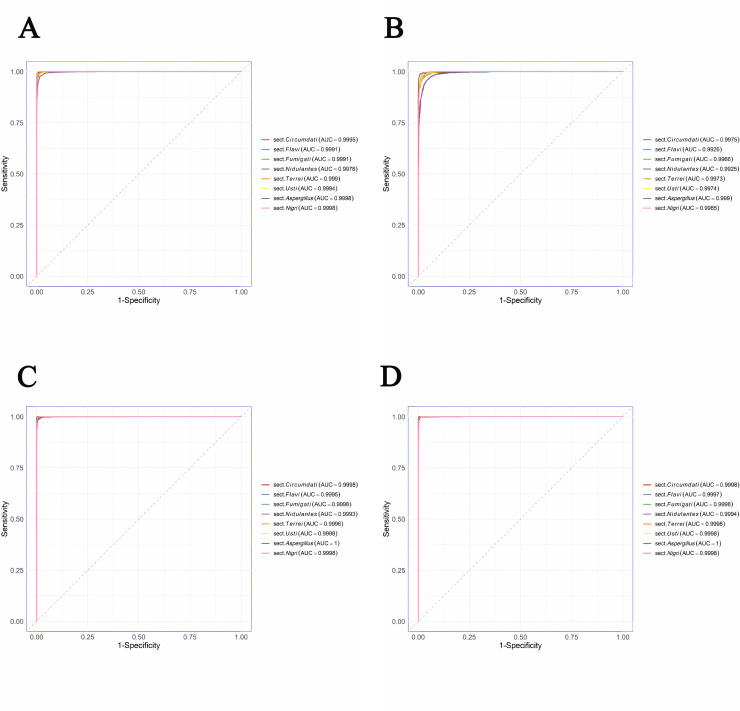
Comparison of ROC curves via the AUC values across four CNN models for classifying eight *Aspergillus* sections. (**A**) GoogleNet, (**B**) ResNet50, (**C**) Xception, (**D**) FungalNet.

This study evaluated the performance of four CNN models for classifying *Aspergillus* strains at the section level. All four models achieved high training accuracies, ranging from 94.4% to 99.8% (Table 3), and each model achieved an area under the curve (AUC) of at least 0.996 (Fig. 2A). Among these four models, FungalNet achieved the best performance across all metrics (AUC: 0.9998, accuracy: 98.45%) (Fig. 3 and 4).

**TABLE 3:** Comprehensive performance comparison of four CNN models at the level of *Aspergillus* section

Model	Accuracy (%)	Precision (%)	Recall (%)	Micro F1-score (%)
GoogleNet	96.66	96.82	96.55	96.68
ResNet50	93.30	92.94	92.50	92.72
Xception	98.21	98.16	98.00	98.08
FungalNet	98.45	98.48	98.23	98.36

To evaluate the discriminative capabilities of four models for each individual *Aspergillus* section, three evaluation metrics (accuracy, precision, specificity), ROC curves, and confusion matrices were analyzed, which demonstrated that Xception and FungalNet achieved similar classification accuracy (>95.90%) for each *Aspergillus* section (Table S1). Additionally, all models demonstrated the highest performance in identifying section *Aspergillus* (AUC: 0.999–1.000, accuracy: 95.80%–99.90%), while the classification metrics were slightly lower for section *Flavi* (AUC: 0.9926–0.9997, accuracy: 86.22%–96.66%) and section *Nidulantes* (AUC: 0.9925–0.9994, accuracy: 88.74%–96.95%) (Fig. 3 and 4).

#### Classification of *Aspergillus* at the species level

To confirm whether different CNN models can discriminate between different *Aspergillus* strains at the species level, the same method was used as at the section level. The results demonstrated that four models exhibited slightly lower performance at the species level compared to the section level. Although the accuracies of four models decreased with the subdivision of the data sets, they maintained strong diagnostic capability (AUC: 0.9962–0.9997, accuracy: 90.29%–97.85%) (Fig. 2B; Table 4). Moreover, FungalNet consistently achieved the best performance in identifying *Aspergillus* species (AUC: 0.9997, accuracy: 97.85%).

**TABLE 4:** Comprehensive performance comparison of four CNN models at the level of *Aspergillus* species

Model	Accuracy (%)	Precision (%)	Recall (%)	Micro F1-score (%)
GoogleNet	95.36	95.39	95.25	95.32
ResNet50	90.29	90.07	90.14	90.10
Xception	97.32	97.33	97.30	97.32
FungalNet	97.85	97.87	97.82	97.84

**Fig 5:**
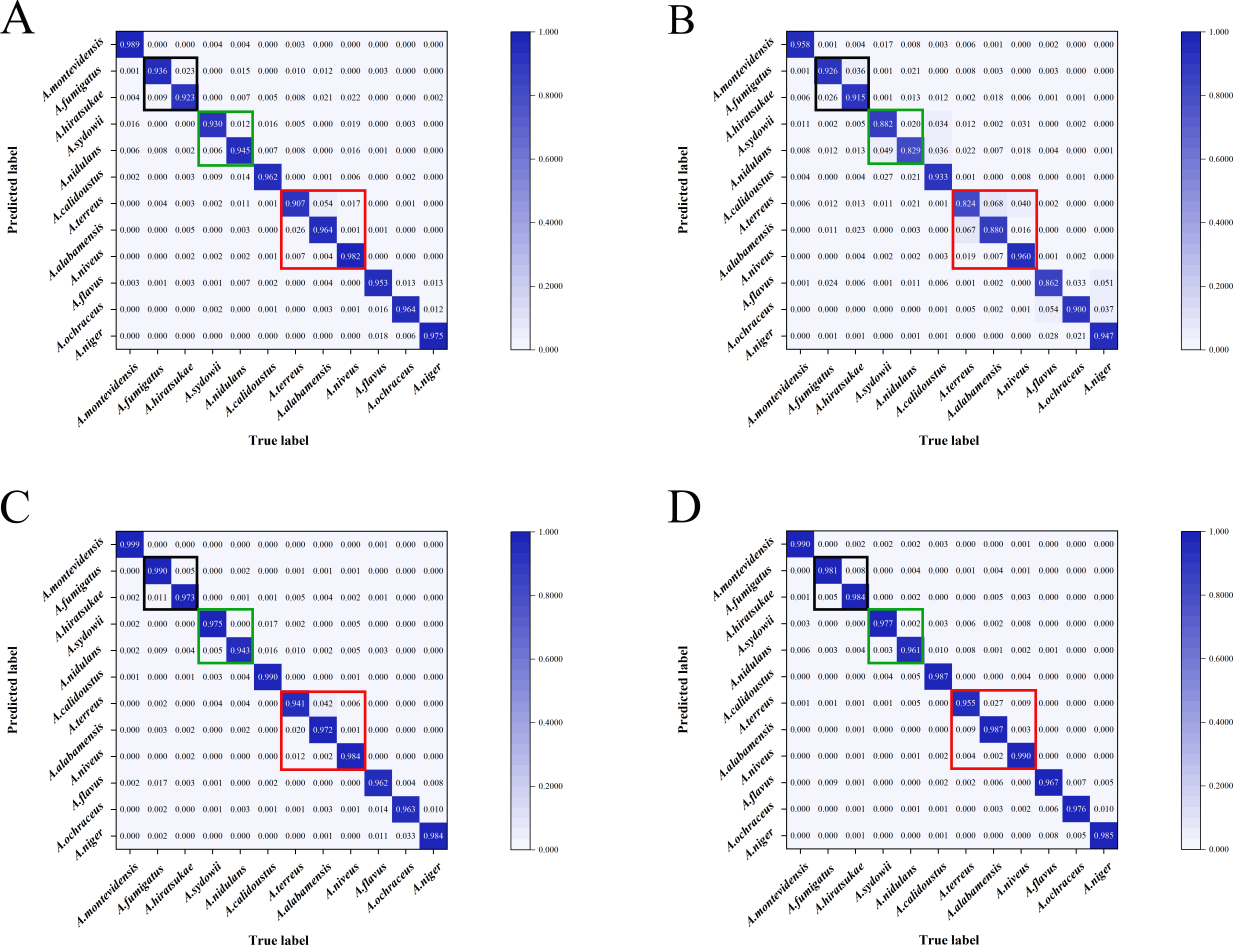
Confusion matrices of four CNN models for *Aspergillus* at the species level (12 species). Three *Aspergillus* sections containing several different *Aspergillus* species are enclosed in boxes of three different colors. Each row represents an instance in the true class and each column represents an instance in the predicted class. The diagonal line shows the prediction accuracy of the CNN model for different *Aspergillus* species. (**A**) GoogleNet, (**B**) ResNet50, (**C**) Xception, (**D**) FungalNet.

**Fig 6:**
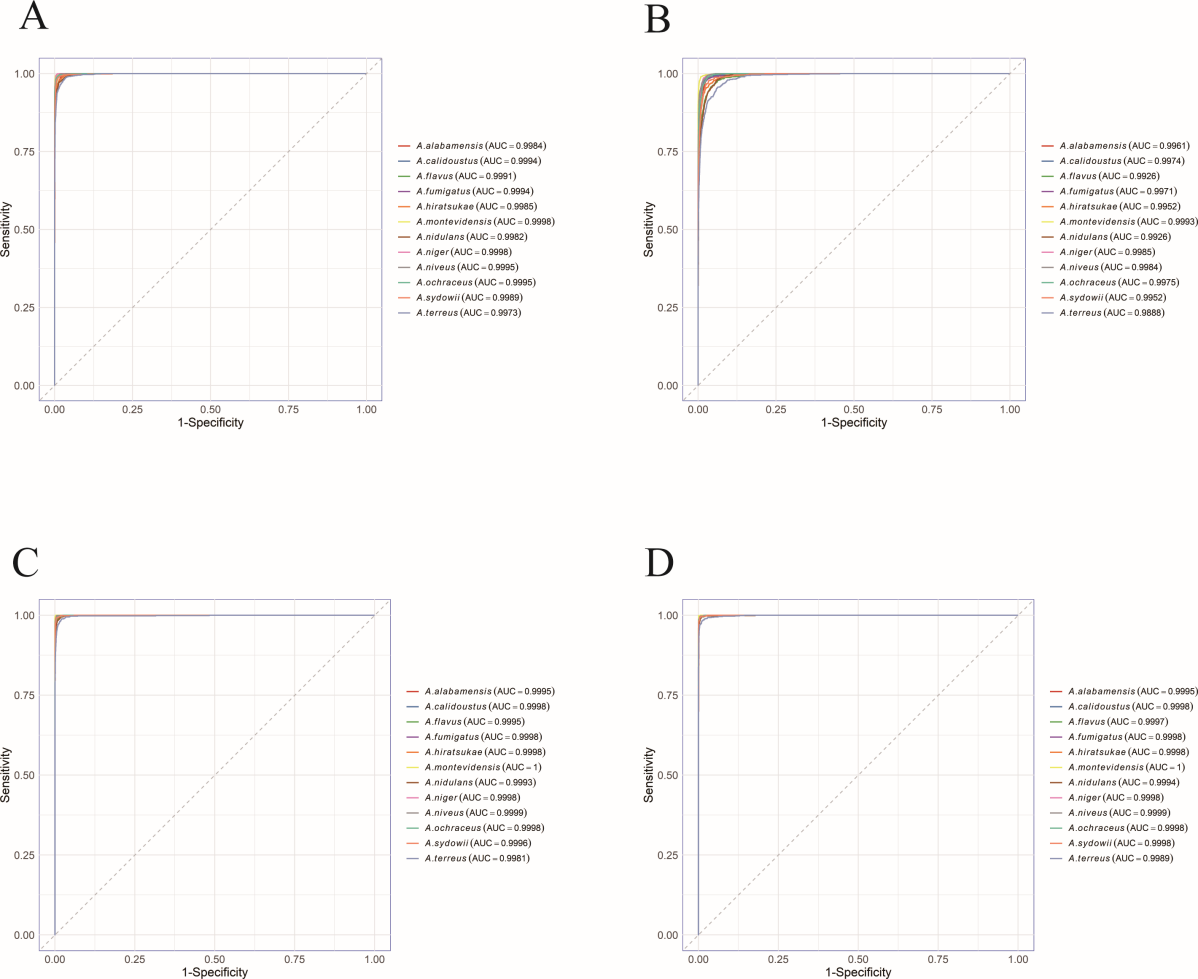
Comparison of ROC curves via the AUC values across four CNN models for classifying 12 *Aspergillus* species. (**A**) GoogleNet, (**B**) ResNet50, (**C**) Xception, (**D**) FungalNet.

As for the discriminative capabilities of four models for each individual *Aspergillus* species, the results demonstrated that Xception and FungalNet also achieved similar classification accuracy (>94.10%) for each *Aspergillus* species (Table S2). Additionally, ROC curve and confusion matrix analyses (Fig. 5 and 6) demonstrated that all four models displayed the encouraging potential for identifying *A. montevidensis* and *A. niveus*. Specifically, Xception achieved the highest classification performance for *A. montevidensis*, with near-perfect accuracy (99.90%) and an AUC score of 1.00, while FungalNet was better at distinguishing *A. niveus*, achieving 99.00% accuracy and a maximal AUC score of 1.00. Meanwhile, all four models displayed slightly reduced performance in identifying *A. terreus*.

## DISCUSSION

A comparative analysis of various *Aspergillus* identification methods (Table 5) reveals that current techniques still face significant limitations. Traditional morphology is cost-effective but labor-intensive and highly subjective; molecular methods provide accurate species identification but are time-consuming and expensive (32); MALDI-TOF requires substantial investment and has database limitations (33). In contrast, the proposed AI-based approach shows strong potential for rapid species-level identification. While maintaining the cost advantages of traditional morphology-based methods, it achieves an identification accuracy approaching molecular techniques, making it particularly suitable for clinical settings that demand both efficiency and reliability.

**TABLE 5:** Comparison of major methods for *Aspergillus* identification^*a*^

Method	Cost-benefit analysis	Time efficiency	Identification level
Traditional morphology	**Low cost. Benefit is operator-dependent**; inexperienced staff may cause misidentification	**Moderate to slow**. Requires culture (2–7 days), followed by microscopic examination	Usually to the **section/complex level**
Molecular methods (PCR/sequencing)	**High cost** for reagents, equipment, and sequencing services. **High benefit**; provides definitive species identification, guiding targeted therapy	**Slow**. The process (culture, DNA extraction, PCR, and sequencing) typically takes **several days to a week**	Accurate identification to the **species level**
MALDI-TOF MS	**Very high initial instrument cost**. Moderate per-test cost. **High benefit**, but identification capability heavily depends on database completeness	**Fast**. After culture, sample processing and analysis can be completed in **minutes**	Usually to the **genus/species level**
AI-based morphology (this study)	**High initial development cost**, but **very low application cost**. Promises **high benefit** by providing standardized, objective, and rapid screening	**Fast**. After culture, AI model analysis takes only **seconds**	Aims for **species-level** identification

^*a*^ Bold text indicates the specific metrics compared across methods: cost-benefit analysis (financial vs. diagnostic value), time efficiency, and identification level.

However, previous studies that employed CNN models to classify *Aspergillus* spp. have generally used lactophenol cotton blue-stained microscopic images with 40× magnification. Although this method continues to serve as the standard for the microscopic identification of fungi, the morphological features among different *Aspergillus* species at 40× magnification are insufficient for accurate species-level identification. In contrast, microscopic images with 100× magnification can reveal more detailed and rich features of *Aspergillus*, particularly the structural characteristics of conidial heads. These features are necessary for the identification of *Aspergillus* species that share highly similar morphology. Furthermore, the results demonstrated that ResNet-50 and Xception exhibited improved performance on our image data set (accuracy: 90.29% and 97.32%, respectively) compared to previous studies utilizing 40× magnification image data sets (accuracy: 81.2% and 84.5%, respectively) (20). Thus, microscopic images with 100× magnification show great potential for precisely identifying *Aspergillus* species.

When using CNN models for classification tasks, the image data quality is crucial for the performance of the model. Therefore, it is imperative to rapidly and effectively enhance the overall quality of the image data set. This study adopted a new approach combining fivefold cross-validation with expert-guided manual selection to ensure the image data quality. This method quickly identified 591 incorrectly predicted images, and 311 low-quality images of them were confirmed and excluded by senior clinical microbiologists. After data preprocessing, four models were trained and validated on the final curated image data set. The results showed that all of the models demonstrated excellent recognition capabilities.

Several phylogenetically related *Aspergillus* species—especially those within the same section, series, and complex (e.g., *A. terreus* and *A. alabamensis*, both belonging to the *Terrei* section, *Terrei* series, and *A. terreus* complex)—share highly similar morphological characteristics due to the conservative traits retained from their common evolutionary ancestor, leading to misidentification even by experienced experts (34, 35). To address these challenges, this study incorporated three different *Aspergillus* sections, each containing several phylogenetically related species. The results showed that all four models demonstrated robust performance in identifying these phylogenetically related species, such as *A. terreus*, *A. alabamensis*, and *A. niveus* (Terrei section); *A. fumigatus* and *A. hiratsukae* (*Fumigati* section); *A. sydowii* and *A. nidulans* (*Nidulantes* section). These results further confirm the potential of combining high-quality data sets with deep learning to distinguish phylogenetically related *Aspergillus* species with high accuracy.

There are emerging reports of rare *Aspergillus* species, such as *A. montevidensis* (36), *A. calidoustus* (37), and *A. niveus* (38), causing invasive aspergillosis in humans. However, the available antifungal susceptibility data are limited and inconsistent due to their low prevalence in clinical settings (25). Furthermore, as indicated by existing reports, these rare species may exhibit elevated resistance to conventional antifungal therapies (39, 40). Thus, the accurate and rapid identification of these rare species has significant clinical implications for aspergillosis diagnosis and antifungal therapy. This study expanded the clinical utility of deep learning in *Aspergillus* morphological analysis by incorporating several rare species (*A. montevidensis, A. alabamensis, A. sydowii, A. calidoustus, A. niveus, and A. hiratsukae*). All four models demonstrated promising identification performance, particularly for *A. montevidensis* (accuracy: 95.80%–99.90%, AUC: 0.9993–1.0000) and *A. niveus* (accuracy: 99.62%–99.91%, AUC: 0.9984–0.9999).

Furthermore, we augmented the image data set with sexual reproductive structures by incorporating four *A. nidulans* strains capable of producing Hülle cells. During validation, FungalNet exhibited robust performance in recognizing these structures, achieving a 93.85% accuracy rate for Hülle cell identification: 168 out of 179 Hülle cell-containing images were correctly identified, with only 11 misclassifications. In the future, the training set will be expanded to include a broader range of *Aspergillus* species that produce sexual structures, thereby continuously enhancing identification performance across species.

This study is also subject to some limitations. Firstly, although the four models already exhibit high accuracy in identifying 12 *Aspergillus* species, we also identified unexpected misclassification between *A. ochraceus, A. niger, and A. flavus*. This phenomenon likely stems from morphological similarities during specific developmental stages—both *A. flavus* and *A. ochraceus* display hemispherical heads and round vesicles, which resemble the juvenile morphology of *A. niger*. Secondly, the restricted number of rare strains included in both the training and validation data sets may have a negative impact on the model’s performance. Future studies incorporating more microscopic images of clinical strains are expected to enhance the robustness and overall classification performance of the model. Moreover, this study is in its preliminary validation stage and lacks an independent clinical data set for robust evaluation of the model’s generalization capability. Thus, we plan to collect additional clinical specimens to construct an independent validation data set.

This study proposes FungalNet, a reliable assistive tool designed for accurately and rapidly identifying *Aspergillus* at both the section and species levels, further demonstrating the potential utility of AI-driven image recognition technology for fungal identification. Nevertheless, real-world applications are still restricted and demand further investigations. In the future, the incorporation of multi-point data and multi-center clinical validation, the expansion of the fungal sample repository to include other *Aspergillus* species and clinically significant filamentous fungi (such as *Fusarium* and *Mucor*) will significantly enhance the model’s applicability. Moreover, the development of an intelligent diagnostic platform that integrates automated scanning and image acquisition devices will improve identification performance and alleviate the shortage of specialized personnel in primary hospitals. These enhancements have the potential to make this technology a standard laboratory diagnostic instrument, thus advancing the clinical prevention and treatment of aspergillosis.

## Data Availability

The data sets used and analyzed during the current study are available from the corresponding author upon reasonable request. The ITS, partial BenA, and partial CaM sequences of the *Aspergillus* strains included in this study have been deposited in GenBank under accession numbers PX645531–PX779386, which are provided in Table S3.

## SUPPLEMENTAL MATERIAL

Table S1 jcm.00012-26-s0001.xlsx Performance metrics of four CNN models compared for classifying 8 *Aspergillus* sections with five-fold stratified cross-validation.

Table S2 jcm.00012-26-s0002.xlsx Performance metrics of four CNN models compared for classifying 12 *Aspergillus* species with fivefold stratified cross-validation.

Table S3 jcm.00012-26-s0003.xlsx Taxonomic, morphological, and molecular characteristics of *Aspergillus* strains included in this study.

ASM does not own the copyrights to Supplemental Material that may be linked to, or accessed through, an article. The authors have granted ASM a non-exclusive, world-wide license to publish the Supplemental Material files. Please contact the corresponding author directly for reuse.
